# Camptodactyly and Early‐Onset Scoliosis in Snijders Blok–Campeau Syndrome

**DOI:** 10.1155/crig/9968980

**Published:** 2026-03-18

**Authors:** Masaki Miura, Yu Kobayashi, Moemi Hojo, Kei Yamada, Hitomi Fujii, Keiko Eimori, Kumiko Yanagi, Tadashi Kaname, Keisuke Nagasaki, Hiromi Nyuzuki, Takeshi Ikeuchi, Jun Tohyama

**Affiliations:** ^1^ Department of Child Neurology, National Hospital Organization Nishiniigata Chuo Hospital, 1-14-1 Masago Nishi-ku, Niigata, 950-2085, Japan; ^2^ Department of Pediatric Orthopedic Surgery, National Hospital Organization Nishiniigata Chuo Hospital, 1-14-1 Masago, Niigata, 950-2085, Japan; ^3^ Department of Genome Medicine, National Center for Child Hearth and Development, 2-10-1 Okura Setagaya-ku, Tokyo, 157-8535, Japan; ^4^ Department of Pediatrics, Niigata University Medical and Dental Hospital, 1-754 Asahimachi-dori Chuo-ku, Niigata, 951-8520, Japan, niigata-u.ac.jp; ^5^ Center for Medical Genetics, Niigata University Medical and Dental Hospital, 1-754 Asahimachi-dori Chuo-ku, Niigata, 951-8520, Japan, niigata-u.ac.jp

**Keywords:** camptodactyly, CHD3, macrocephaly, onset scoliosis, Snijders Blok–Campeau syndrome

## Abstract

Snijders Blok–Campeau syndrome is caused by a heterozygous *CHD3* pathogenic variant, and characterized by features including macrocephaly, speech delay, hypotonia, and joint laxity. In the hereby‐presented case, a Japanese girl with Snijders Blok–Campeau syndrome displayed bilateral middle finger camptodactyly and early‐onset scoliosis, beyond the characteristic symptoms such as intellectual disability, facial abnormalities, macrocephaly, and hypotonia due to a recurrent *CHD3* pathogenic variant. This case expands our knowledge of the phenotypic spectrum of Snijders Blok–Campeau syndrome.

## 1. Introduction

Snijders Blok–Campeau syndrome (SNIBCPS) (OMIM #618205) is an autosomal dominant neurodevelopmental disorder caused by a heterozygous *CHD3* pathogenic variant [1]. *CHD3* encodes an ATP‐dependent chromatin remodeling protein, critically involved in neurodevelopmental processes [2]. Blok et al. initially identified a cohort of 35 patients with the *CHD3* pathogenic variant [1]. Drivas et al. included 24 patients in their second cohort [3]. Mizukami et al. described the first Japanese patient with SNIBCPS [4]. To date, more than 100 cases have been reported worldwide [1, 3–10], and the main features of this syndrome comprise global developmental delay, intellectual disability, macrocephaly, speech delay, hypotonia, and joint laxity. However, the correlation between SNIBCPS genotype and phenotype remains unclear. In this study, we report a case of a Japanese girl with SNIBCPS and the recurrent *CHD3* variant presented with bilateral middle finger camptodactyly and early‐onset scoliosis (EOS). Camptodactyly is a novel result, and EOS is less common in SNIBCPS, which expands the phenotypic spectrum of SNIBCPS.

## 2. Case Presentation

A 9‐year‐old Japanese girl with nonconsanguineous healthy parents was presented at our institution with no family history of congenital anomalies. Her antenatal period was uneventful, and she was born at term via vaginal delivery without complications. Her birth weight, length, and head circumference were 2774 g (−0.69 standard deviation [SD]), 48.5 cm (−0.27 SD), and 34.5 cm (+0.95 SD), respectively. The patient was referred to our orthopedic department with a suspected left hip dislocation at the age of 6 months. Orthopedic assessments documented normal anatomy, and the patient was instructed to undergo regular follow‐ups.

She was referred to our hospital for investigation of delayed head control, macrocephaly, and bilateral middle finger camptodactyly at the age of 7 months. At that time, her height, weight. and head circumference were 67.3 cm (+0.47 SD), 7.77 kg (+0.52 SD), and 46 cm (+3.33 SD), respectively. Physical examination revealed hypertelorism, a wide nasal bridge, cryptotia, and scoliosis. Flexion contracture of both middle fingers was observed, and the extension of both index fingers was slightly restricted as well. Static finger splints were used for camptodactyly. The EOS of the patient posed a risk of significant progression. Therefore, the patient was instructed to wear a thoracic brace, and careful observation was started. The patient exhibited pes planus with valgus. Although her hypotonia persisted, the scoliosis and pes planus with valgus did not progress. Camptodactyly improved, and fingers could be extended at 9 years of age.

This patient has exhibited moderate developmental delay since the initial visit, which has persisted thereafter. Moderate motor and speech delays were present, with walking achieved at 2 years and 6 months and first speech at 3 years and 5 months. At the age of 9 years, the patient exhibited a moderate‐to‐severe intellectual disability, inattentive hyperactivity, and slight autistic features. However, she displayed no seizures. She was able to speak a few words and communicate with others. The patient occasionally exhibited emotional dysregulation and tantrums when she could not communicate her thoughts properly. As determined using the Tanaka−Binet test, the intelligence quotient of the patient at the age of 9 years was 38, confirming a severe intellectual disability.

## 3. Cerebral Imaging and Radiograph

Brain magnetic resonance imaging revealed reduced cerebral volume and mildly delayed myelination (Figure 1(a)). The radiographs of both hands displayed camptodactyly of the proximal interphalangeal (PIP) joints (Figure 1(b)). In addition, spinal radiography indicated scoliosis with a major curve Cobb angle of 25° (Figure 1(c)). However, computed tomography images revealed no vertebral anomalies.

**FIGURE 1:**
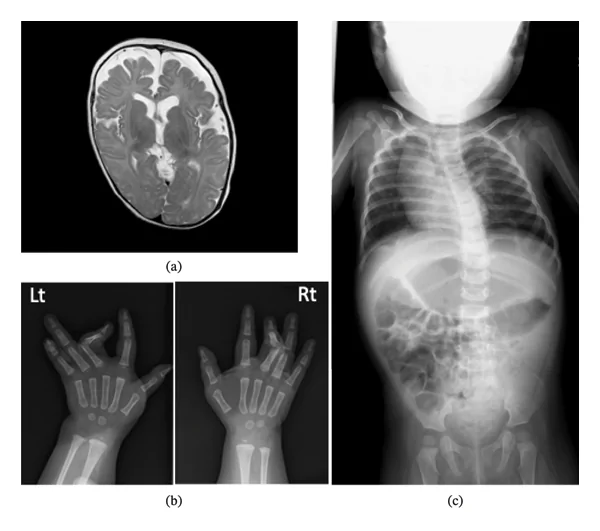
Axial T2‐weighted brain magnetic resonance imaging (MRI) scan and hand and spine X‐ray of the patient, obtained at the age of 7 months. (a) Brain MRI revealing decreased cerebral volume and mildly delayed myelination. (b) Radiographs of both hands displaying a flexion deformity in the bilateral middle finger at the proximal interphalangeal (PIP) joint. Lt: left; Rt: right. (c) Posteroanterior spine radiograph presenting thoracolumbar left curvature with a Cobb angle of 25°.

## 4. Metabolic and Cytogenetics

Neonatal metabolic screening results were optimal. Metabolic screening at 7 months displayed no abnormalities, and G‐band karyotyping revealed a normal 46, XX karyotype.

## 5. Genetic Analysis

Since the cause of these symptoms remained unknown, trio‐based whole‐exome sequencing of the patient and her parents was performed at the age of 2.5 years, revealing a *de novo* heterozygous missense variant, NM_00100273.3:c.3505C > T:p.(Arg1169Trp) in *CHD3*, a recurrent variant [1, 10]. Finally, the variant was confirmed by Sanger sequencing, and in silico prediction tools suggested the variant had a deleterious nature (CADD score: 25.7; PolyPhen‐2.0: probably damaging; SIFT: deleterious). According to the ACMG/AMP guidelines [11], the variant was classified as pathogenic (PS1, PS2, PM2, PP1, and PP3). Clinical and molecular genetic studies were performed in accordance with the Declaration of Helsinki and approved by the Institutional Review Board of Niigata University School of Medicine. Written informed consent was obtained from the parents of the patient.

## 6. Discussion

The patient in this study exhibited the characteristic symptoms of intellectual disability, facial abnormalities, macrocephaly, and hypotonia. We compared the clinical features of our patients with those of patients from previous studies (Table 1). Moreover, the hereby‐presented patient displayed camptodactyly, a condition that has not been previously reported related to this disease, and scoliosis has only been reported in a single other patient [8], expanding the phenotypic spectrum of SNIBCPS. LeBreton et al. described central precocious puberty as a rare phenotype of this syndrome [5], and Coursimault et al. reported arachnodactyly, lower limb asymmetry, and renal abnormalities [6]. In addition, van der Spek et al. investigated the phenotypic variation in 21 families with inherited *CHD3* heterozygous variants and discovered that their phenotypes were similar to those previously associated with *de novo CHD3* variants, whereas heterozygous parents were mildly or not affected [7], suggesting that the phenotypic spectrum of SNIBCPS is related to the variable expressivity or reduced *CHD3* penetrance [7].

**TABLE 1:** Phenotype summary of previously reported Snijders Blok–Campeau syndrome cases and comparison between the phenotypes in previous cases involving the c.3505C > T variant and the present case.

	**Combined cohorts** **(90 cases)** ^a^	Snijders Blok et al. [**1**] individual 22	Snijders Blok et al. [**1**] individual 23	Snijders Blok et al. [**1**] individual 24	Tie et al. [**10**] **patient 2**	**The present case**

Age	1 to 59 years	17 years	2 years 8 months	4 years 5 months	11 months	9 years
Sex		Male	Male	Male	Female	Female
Variant in CHD3		c.3505C > T	c.3505C > T	c.3505C > T	c.3505C > T	c.3505C > T
Developmental delay	90/90 (100%)	+	+	+	+	+
Speech delay	87/87 (100%)	+	+	+	+	+
Intellectual disability	61/80 (76%)	−	+	+	+	+
Hypotonia	66/81 (81%)	+	+	−	−	+
Autistic features	34/84 (40%)	−	−	+	−	−
Seizures	10/65 (15%)	−	−	−	−	−
Macrocephaly	39/86 (45%)	+	+	−	+	+
CNS abnormality by MRI	38/74 (51%)	−	+	N/A	+	+
Prominent extra‐axial space, delayed myelination						
Facial dysmorphism						
Frontal bossing	46/86 (53%)	−	+	+	+	−
Ocular hypertelorism	55/85 (65%)	+	+	−	+	+
Broad nasal bridge	41/54 (76%)	N/A	N/A	N/A	+	+
Abnormality of the dentition	8/55 (15%)	−	+	−	−	−
Low set ears	14/34 (41%)	N/A	N/A	N/A	−	+
Visual abnormality	49/87 (56%)	−	+	−	−	−
Congenital heart disease	8/69 (12%)	−	−	−	−	−
Male abnormalities of the genital system	12/40 (30%)	+	+	−	N/A	N/A
Joint laxity	30/84 (36%)	−	+	−	−	+
Abnormalities of the skeletal system	14/45 (31%)	−	+	−	−	+
Other specific features		ADHD, thin and concave nails, thin fingernails, cutaneous syndactyly of toes, inguinal hernia	Abnormal bladder shape on MRI	Inguinal hernia	Elevated serum 25‐hydroxyvitamin D	Camptodactyly, scoliosis

Abbreviations: ADHD, attention deficit hyperactive disorder; CNS, central nervous system; MRI, magnetic resonance imaging; N/A, not available.

^a^Combined cohorts from Snijders Blok et al. [1], Drivas et al. [3], Mizukami et al. [4], Coursimault et al. [5], LeBreton et al. [6], van der Spek et al. [7], Gao et al. [8], Chen et al. [9], and Tie et al. [10].

The c.3505C > T variant identified in the hereby‐presented patient was a recurrent variant located in a hotspot within the helicase C‐terminal domain of the CHD3 protein [1]. We compared the phenotypes of previously reported patients with the c.3505C > T variant [1, 10] and that of the hereby‐described patient, and then investigated the genotype−phenotype relationship (Table 1). Patients with the c.3505C > T variant exhibited a lower percentage of autism symptoms, seizures, and visual abnormalities compared to the overall patient population, and a relatively higher percentage of macrocephaly. All five patients displayed developmental and speech delays. However, one of them retained borderline intelligence. No patients developed seizures. The incidences of other symptoms (such as hypotonia, joint laxity, or abnormalities of the skeletal system) varied. Therefore, delineating specific genotype−phenotype correlations of this variant would be difficult [7, 8]. SNIBCPS phenotypes could be potentially affected by further genetic factors, such as a second hit on another allele or the involvement of other pathogenic genes [7].

In this study, camptodactyly was a novel feature related to this condition. Camptodactyly is a nontraumatic and progressive flexion contracture of the PIP joint, with usually correctable flexion deformities, that occurs in less than 1% of the population. All structures at the base of the finger are involved in the pathogenesis of the deformity [12]. The disease is classified into three types, i.e., infantile, adolescent, and syndromic [13]. More than 60 chromosomal abnormalities or gene mutations are associated with syndromic camptodactyly, including 22q11 deletion, dup (15) (q24q26.3), trisomy 18 syndrome, and Loeys–Dietz syndrome [12, 14]. Syndromic camptodactyly often affects multiple fingers, and bilateral deformities with severe contractures are common [12]. In this patient, although joint incompatibility or tendon abnormalities were the suspected causes of camptodactyly, the exact cause remains unknown. The patient displayed nonprogressive symptoms, and conservative treatment improved the extension range.

Radiography revealed scoliosis in our patient at the age of 7 months. Another patient exhibited scoliosis at the age of 2 years [8]. EOS is defined as any spinal deformity that is present before the age of 10, regardless of etiology [15]. In general, the earlier scoliosis develops, the more severe the spinal deformity [15]. EOS mechanism progression varies depending on its form and etiology [16], the latter being categorized as idiopathic, neuromuscular, congenital, and syndromic [15]. Syndromic deformities are caused by scoliosis‐associated clinically defined patterns (i.e., syndromes) and are not directly attributed to congenital or neuromuscular causes [15]. Deformity progression might result in thoracic insufficiency syndrome, in which the distorted thorax cannot support healthy respiratory function or lung growth [17]. Therefore, understanding the progression characteristics of each disease case is important in determining the EOS treatment policy. We hypothesized here that the EOS was likely due to hypotonia or joint laxity. Although EOS did not progress, careful follow‐up would be necessary to determine whether this syndrome displays a good prognosis.

Macrocephaly is among the characteristics of this syndrome. In numerous genetic disorders, macrocephaly is combined with intellectual disability [18]. Pathogenic variants in nucleosome remodeling and deacetylase complex have been associated with neurodevelopmental disorders (e.g., macrocephaly), similar to other overgrowth and intellectual disability syndromes [2]. The present case supports this phenomenon. Therefore, recognizing SNIBCPS in the differential diagnosis of neurodevelopmental disorders with macrocephaly is important.

In conclusion, we described a patient with SNIBCPS and abnormalities of the skeletal system, specifically bilateral middle finger camptodactyly and EOS, although the genotype−phenotype correlation of SNIBCPS remained unclear. Nevertheless, this case expands our knowledge of the phenotypic spectrum of SNIBCPS.

## Funding

This study was supported by the Initiative on Rare and Undiagnosed Diseases (Grant nos. 16ek0109166, 22ek0109549, and JP25ek0109760) of the Japan Agency for Medical Research and Development (AMED).

## Conflicts of Interest

Tadashi Kaname and Kumiko Yanagi received research grants from the Japan Agency for Medical Research and Development (AMED) related to this study. The remaining authors declare no conflicts of interest.

## Data Availability

The data that support the findings of this study are available on request from the corresponding author. The data are not publicly available due to privacy or ethical restrictions.
